# Copper Smoke and Its Effects
*Report on the Copper Smoke, and the Industrial Diseases of Coppermen. By Thomas Williams, M.D. Swansea: Herbert Jones. 1854.


**Published:** 1857-04

**Authors:** 


### COPPER SMOKE AND ITS EFFECTS.*

Although Dr. Williams's Report has been before the public two years, it has received far less attention than it deserves. It is a work written in clear and forcible language, philosophical without restraint, and scientific without complexity. It is full of strange facts and varied history. We have not space for detail; let us, however, record an opinion: "The *direct* chemical and pathological effects of the copper smoke upon the human body must be carefully separated from those *indirect* consequences, which flow from its destructive and corrosive operation upon the surface vegetation of land. In this way, beyond doubt, unless the entire tendency of modern science be utterly false, a thousand causes of disease are rendered inoperative. If it were possible to maintain a *permanent* infusion of copper smoke in the atmosphere of a given locality, the author records his deliberate belief that the population of such locality would be *permanently* exempt from those epidemic diseases, whose causative germs, whatever they may be in essence, travel and multiply from place to place in the atmosphere." If the author should ever *establish* this fact, he will have thrown a marvellous light on the causes and prevention of epidemics.

* Report on the Copper Smoke, and the Industrial Diseases of Coppermen. By Thomas Williams, M.D. Swansea: Herbert Jones. 1854.

